# Preparation, Characterization and Industrial Application of Nanocellulose

**DOI:** 10.3390/nano13101592

**Published:** 2023-05-10

**Authors:** Marc Delgado-Aguilar, Carlos Negro

**Affiliations:** 1 LEPAMAP-PRODIS Research Group, University of Girona, C. Maria Aurèlia Capmany, 61, 17003 Girona, Spain; 2 Department of Chemical Engineering and Materials, Complutense University of Madrid, Avda. Complutense s/n, 28040 Madrid, Spain

The international research community has made significant efforts in the production, characterization, and application of cellulose nanofibers (CNFs) in many sectors. CNFs have a great ability to create 3D-structured networks without any additional crosslinking. This network-forming capacity, together with excellent mechanical characteristics and a reactive surface, makes CNF a nanomaterial with great opportunities in several markets. The CNF market is expected to grow from USD 297 million in 2020 to USD 783 million by 2025 at a compounded annual growth rate (CAGR) of 21.3%. The term CNF is often used for those cellulose nanofibers where the lignin content is residual or absent. However, CNFs can be also produced from fibers containing lignin, called lignocellulosic nanofibers (LCNFs). Given the complexity of fiber structure, nanocellulose production processes are still under development at large scale and there are still several challenges in terms of characterization that the scientific community will have to face in the coming years.

Several applications for nanocellulose have been reported to date, but some of them are far from ready for industrial implementation. This is due to the lack of robust production and characterization processes and protocols, but also due to the unknown side-effects that may be represented in several industrial operations. However, the literature reports many benefits of using nanocellulose in a myriad of sectors, such as papermaking, composites, biomedicine, environmental applications, and many others.

For all the above, we decided to organize a Special Issue on “Preparation, Characterization and Industrial Application of Nanocellulose”. This Special Issue is the result of 13 contributions from 50 researchers in 22 research groups from 10 countries (Argentina, Canada, China, France, Japan, Portugal, Spain, Sweden, Tunisia, and the United States of America). These contributions cover most of the relevant areas related to nanocellulose production, characterization, and industrial applications.

In this Special Issue, the authors have discussed novel production methods of nanostructured cellulose, as well as alternative raw materials with the purpose of enhancing the sustainable character of this nanomaterial. Some examples are the use of date palm wastes [1], annual plants [2], and harvesting residues [3]. In terms of production methods, innovative systems such as oxalic acid [4] or deep eutectic solvents (DES) [5] have been proposed in this Special Issue, as well as bottom-up approaches such as bacterial cellulose [6]. While these papers report an exhaustive characterization of the developed materials, the Special Issue also counts on specific articles devoted to characterization techniques, such as using gel-point and morphological measurements for assessing the characteristics of nanocellulose [7,8].

This Special Issue also covers relevant aspects related to nanocellulose applications, with a special emphasis on their use as coating agents for paper [3,9,10,11], for the reinforcement of nanocomposites [6], and one research article is devoted to wastewater treatment [12,13].

We would like to acknowledge all the authors, reviewers, and the editorial team from *Nanomaterials* who made the process of publishing a Special Issue an exciting and motivating experience.
